# Effects of Body Mass Index and Body Fat Percent on Default Mode, Executive Control, and Salience Network Structure and Function

**DOI:** 10.3389/fnins.2016.00234

**Published:** 2016-06-14

**Authors:** Chase R. Figley, Judith S. A. Asem, Erica L. Levenbaum, Susan M. Courtney

**Affiliations:** ^1^Department of Radiology, University of ManitobaWinnipeg, MB, Canada; ^2^Biomedical Engineering Graduate Program, University of ManitobaWinnipeg, MB, Canada; ^3^Neuroscience Research Program, Kleysen Institute for Advanced Medicine, Winnipeg Health Sciences CentreWinnipeg, MB, Canada; ^4^Department of Psychological and Brain Sciences, Johns Hopkins UniversityBaltimore, MD, USA; ^5^Department of Neurobiology and Behavior, University of CaliforniaIrvine, CA, USA; ^6^Center for the Neurobiology of Learning and Memory, University of CaliforniaIrvine, CA, USA; ^7^School of Medicine and Dentistry, University of Rochester Medical CenterRochester, NY, USA; ^8^Solomon H. Snyder Department of Neuroscience, Johns Hopkins UniversityBaltimore, MD, USA; ^9^F. M. Kirby Research Center for Functional Brain Imaging, Kennedy Krieger InstituteBaltimore, MD, USA

**Keywords:** brain, cognition, functional connectivity, gray matter, neuroimaging, obesity, structural connectivity, white matter

## Abstract

It is well established that obesity decreases overall life expectancy and increases the risk of several adverse health conditions. Mounting evidence indicates that body fat is likely also associated with structural and functional brain changes, reduced cognitive function, and greater impulsivity. However, previously reported differences in brain structure and function have been variable across studies and difficult to reconcile due to sample population and methodological differences. To clarify these issues, we correlated two independent measures of body composition—i.e., body mass index (BMI) and body fat percent (BFP)—with structural and functional neuroimaging data obtained from a cohort of 32 neurologically healthy adults. Whole-brain voxel-wise analyses indicated that higher BMI and BFP were associated with widespread decreases in gray matter volume, white matter volume, and white matter microstructure (including several regions, such as the striatum and orbitofrontal cortex, which may influence value assessment, habit formation, and decision-making). Moreover, closer examination of resting state functional connectivity, white matter volume, and white matter microstructure throughout the default mode network (DMN), executive control network (ECN), and salience network (SN) revealed that higher BMI and BFP were associated with increased SN functional connectivity and decreased white matter volumes throughout all three networks (i.e., the DMN, ECN, and SN). Taken together, these findings: (1) offer a biologically plausible explanation for reduced cognitive performance, greater impulsivity, and altered reward processing among overweight individuals, and (2) suggest neurobiological mechanisms (i.e., altered functional and structural brain connectivity) that may affect overweight individuals' ability to establish and maintain healthy lifestyle choices.

## Introduction

Obesity affects more than 850 million people (i.e., 10% of men and 14% of women) worldwide (Finucane et al., 2011), is a major risk factor for many serious health conditions—including type II diabetes (Kahn et al., 2006), cardiovascular disease (Hubert et al., 1983), hypertension (Rahmouni et al., 2005), sleep apnea (Vgontzas et al., 1994), and cancer (Calle and Kaaks, 2004)—and is thought to be one of the greatest contributors to preventable death in the United States (Flegal et al., 2005; Jia and Lubetkin, 2010). Studies of childhood, teenage, and adult obesity have also provided consistent evidence that higher body mass index (BMI)^1^ is associated with a variety of cognitive deficits (Elias et al., 2003, 2012; Cournot et al., 2006; Waldstein and Katzel, 2006; Boeka and Lokken, 2008; Nilsson and Nilsson, 2009; van den Berg et al., 2009; Gunstad et al., 2010; Verdejo-García et al., 2010; Mobbs et al., 2011; Smith et al., 2011; Gustafson, 2012; Sellbom and Gunstad, 2012), and may even lead to early-onset dementia (Whitmer et al., 2008; Naderali et al., 2009). However, despite the myriad of obesity-related physical and mental health concerns, most overweight individuals struggle to initiate and maintain behaviors that would reduce adiposity and improve their overall health (e.g., Wadden et al., 2004). It has been suggested that body composition itself might somehow affect the neural systems that underlie cognition, motivation, self-control, and salience processing, which would in turn affect one's ability to make better lifestyle choices (e.g., forgoing immediate and/or highly salient rewards for the sake of longer-term health and wellness goals). Nonetheless, although many studies have attempted to address this question, the results to date have been variable and inconclusive.

Elevated BMI has been linked to reduced total brain volumes among healthy adolescents and middle-aged adults (Ward et al., 2005; Gunstad et al., 2008; Debette et al., 2010; Cazettes et al., 2011; Bobb et al., 2014), and to an accelerated rate of age-related cortical atrophy (Driscoll et al., 2012). Several neuroimaging studies have also reported localized gray matter volume decreases in the hippocampus and parahippocampal cortex (Jagust et al., 2005; Taki et al., 2008; Raji et al., 2010; Walther et al., 2010; Bobb et al., 2014), dorsolateral and medial prefrontal cortices (Pannacciulli et al., 2006; Taki et al., 2008; Walther et al., 2010; Brooks et al., 2013), cerebellum (Pannacciulli et al., 2006; Taki et al., 2008; Walther et al., 2010), cuneus/precuneus (Taki et al., 2008; Walther et al., 2010), orbitofrontal cortex (Raji et al., 2010; Maayan et al., 2011), dorsal striatum (i.e., caudate and putamen) (Raji et al., 2010), thalamus (Raji et al., 2010), and brainstem (Taki et al., 2008; Walther et al., 2010). One study has reported gray matter increases (rather than decreases) in the orbitofrontal cortex, nucleus accumbens, putamen, and hypothalamus (Horstmann et al., 2011).

Fewer studies have examined relationships between body composition and white matter volumes, and the findings to date have been relatively inconsistent. Although early reports indicated that both regional (Pannacciulli et al., 2006) and global (Haltia et al., 2007) white matter volumes were increased among obese participants, more recent studies have either found no significant relationship between body composition and white matter volume (Gunstad et al., 2008; Bobb et al., 2014), or that higher BMI was associated with widely distributed white matter atrophy (Karlsson et al., 2013). In addition to these macrostructural changes, a small number of diffusion tensor imaging (DTI) studies have associated higher BMI with reduced white matter microstructure in the corpus callosum (Mueller et al., 2011; Stanek et al., 2011; Verstynen et al., 2012; Karlsson et al., 2013; Xu et al., 2013), cerebellar white matter (Verstynen et al., 2012), internal capsule (Verstynen et al., 2012; Karlsson et al., 2013), and corticospinal tract (Karlsson et al., 2013). However, at least one study (Verstynen et al., 2012) has reported positive correlations among regions of the frontopontine tract, inferior longitudinal fasciculus (ILF), middle and superior cerebellar peduncles, and the splenium of the corpus callosum.

Growing attention has also been placed on examining obesity-related metabolic and functional connectivity alterations. Recent studies in this domain have shown that higher BMI is associated with lower resting glucose metabolism (Volkow et al., 2009) and decreased cerebral blood flow (Willeumier et al., 2011) throughout the prefrontal and cingulate cortices, and that levels of baseline metabolism in these regions are correlated with executive function and working memory performance (Volkow et al., 2009). Electrophysiology data have suggested that overweight participants have increased whole-brain synchrony in the delta (0.5–4 Hz) and beta (13–30 Hz) frequency bands compared to lean individuals (Olde Dubbelink et al., 2008), and resting state functional magnetic resonance imaging (rs-fMRI) results suggest that these connectivity differences might be network-specific—although there are conflicting reports as to which regions and networks are involved.

One fMRI study reported that obesity was linked to increased functional connectivity in the default mode network (DMN) and reduced functional connectivity of the insula (Kullmann et al., 2012), which is associated with reward processing, emotional control, and serves as the primary hub of the salience network (SN) (Seeley et al., 2007; Hampshire et al., 2010). However, another study found that obesity was associated with significantly increased functional connectivity of the putamen and marginally increased connectivity of the insula, amygdala, and superior parietal lobule (García-García et al., 2013). Finally, despite the preponderance of evidence regarding obesity and decreased cognitive performance, to the best of our knowledge, no previous studies have directly examined the relationship between body composition and functional connectivity throughout the executive control network (ECN), which includes parietal and prefrontal regions that are involved in attention, working memory, and other aspects of high-level cognitive processing (Seeley et al., 2007).

One common thread among the aforementioned studies, and obesity literature in general, is the routine use of BMI as the primary (and often only) measure of body composition. BMI is non-invasive, easy to acquire, and widely accepted as a clinical standard. However, because it is not able to distinguish between lean and adipose tissue, a number of studies have cautioned that it is, at best, an indirect measure of actual fat content and, at worst, a relatively poor measure of metabolic health (Romero-Corral et al., 2008; Jackson et al., 2009; Krakauer and Krakauer, 2012; Ahima and Lazar, 2013)—particularly on an individual basis or in small sample sizes. As a result, the use of BMI measures alone (i.e., as a surrogate for body fat) may account for some of the inconsistencies between previous neuroimaging studies. Alternatively, bioelectrical impedance analysis (BIA) scales are now widely available, highly portable, minimally invasive, relatively inexpensive, and offer another quantitative method for estimating body composition with high inter-rater reliability (Kushner and Schoeller, 1986; Segal et al., 1988; Kushner, 1992). BIA measures the electrical impedance of body tissues with small (i.e., < 1 mA) alternating currents, in which impedance is a function of tissue resistance and the reactance (i.e., the inductance and capacitance) of membranes, interfaces, and nonionic tissues (Kyle et al., 2004a,b). Estimates of body fat then employ complex multicomponent models, which rely on a few basic assumptions (e.g., that the density of each participant's fat free mass is consistent with predetermined age-, gender-, and height-specific empirical values, and that hydration levels are relatively consistent within and across individuals).

Understanding the specific neural systems that are associated with high body fat will help to explain obesity-related cognitive and behavioral differences and hopefully pave the way to treatment and prevention. Thus, the goal of the current study was to identify the primary neural correlates of adiposity using convergent evidence from multiple structural and functional neuroimaging methods. Specifically, we collected rs-fMRI, high-resolution T_1_-weighted structural imaging, and DTI data from 32 healthy adult volunteers. We then investigated how two different measures of body composition—BMI and body fat percent (BFP)—were related to voxel-wise changes in gray matter volume, white matter volume, and white matter microstructure throughout the entire brain. We then went on to examine how individual differences in body composition (i.e., BMI and BFP) were related to resting state functional connectivity, white matter volume, and white matter microstructure throughout the DMN, ECN, and SN. To the best of our knowledge, this is the first human neuroimaging study allowing: (1) direct comparisons between adiposity, regional brain volume, white matter microstructure, and functional connectivity within the same sample population, and (2) cross-validation of these results using both conventional BMI measurements and bioelectric impedance-based estimates of body composition.

## Materials and methods

### Study participants

**Figure 1:**
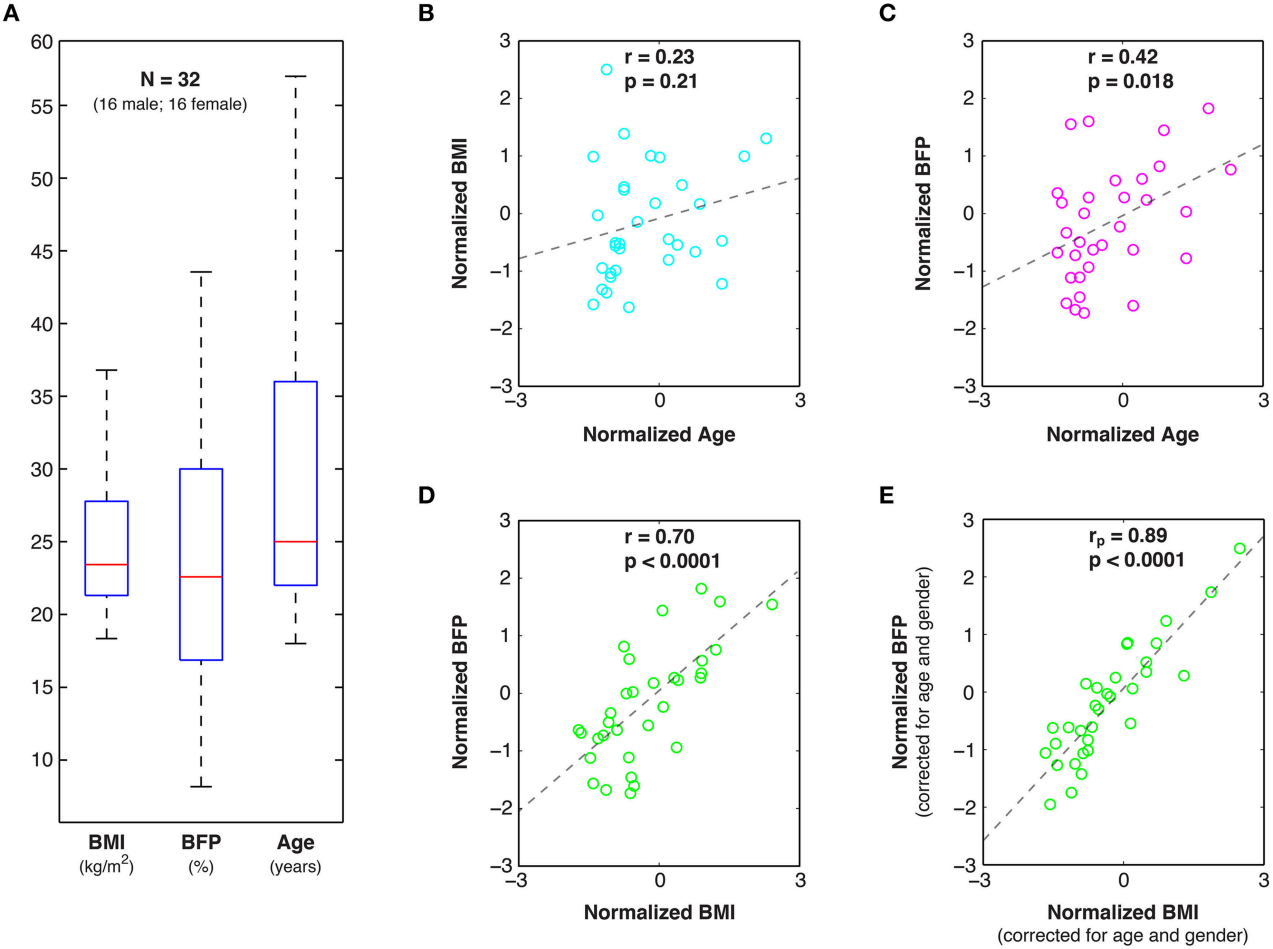
**Age and body composition data. (A)** Box and whisker plots of Body Mass Index (BMI), Body Fat Percent (BFP), and Age among the 32 healthy study participants, as well as linear correlations (r) and statistical significance values (*p*) between: **(B)** normalized BMI and Age, **(C)** normalized BFP and Age, and **(D)** normalized BFP and BMI. **(E)** Partial correlations (*r*_*p*_) were also performed between normalized BFP and normalized BMI to factor out the effects of age and gender.

In an effort to span a broad cross-section of the North American population, 32 healthy volunteers (16 male; 16 female) were recruited from the Charles Village, Hampden, and Roland Park neighborhoods in Baltimore, Maryland. All prospective participants were verbally screened to exclude those with any history of neurological injury/disease, psychiatric disorder, metabolic disease (including diabetes), autoimmune disorder, or substance abuse (including alcohol and/or tobacco). Of the 32 subjects, 19 were Caucasian, 9 were Asian, 2 were African-American, and 2 were Hispanic (based on self-report). Age and body composition measures for these participants are shown in Figure 1 and Figure S1. This study was approved by the Johns Hopkins University (JHU) Institutional Review Board, and all participants provided written informed consent prior to enrollment.

### Body composition measurements

BMI and BFP measurements were acquired at two timepoints—i.e., the beginning and end (~2 hours apart)—during each participant's experimental session. Height was measured twice using a wall-mounted height chart (QuickMedical, Issaquah, Washington, USA), and the average of these measurements was rounded to the nearest half centimeter so that the necessary height, age, and gender information could be entered into our BC-350 bioelectric impedance scale (Tanita Corporation of America Inc., Arlington Heights, Illinois, USA). The replicate body composition measurements were then averaged to determine each participant's mean BMI and BFP.

In order to minimize hydration variance across the sample: (1) all participants were asked to avoid caffeine, alcohol, and other diuretics, but otherwise eat and drink normally prior to the study, (2) initial measurements (immediately prior to the MRI portion of the study) were always acquired while participants had an empty bladder, and (3) secondary measurements (immediately following the MRI portion of the study) were always acquired while participants had an unvoided bladder. With our bioelectric impedance scale, it was not possible to control for built-in assumptions regarding fat-free mass densities, which can introduce systematic bias across ethnic groups (Wagner and Heyward, 2000)—although it is worth noting that this criticism also applies to BMI and hydrostatic densitometry measures.

Nevertheless, despite these assumptions, BIA has several advantages. It requires minimal training, can be easily standardized to reduce inter-rater variability, and has been shown to provide highly consistent estimates of body composition compared to skin-fold measurements or air displacement plethysmography, even in consumer-level models (Peterson et al., 2011). At the same time, BIA offers a less invasive, less expensive, and more portable alternative to “criterion standard” body composition analysis methods such as hydrostatic densitometry or dual-energy X-ray absorptiometry (DEXA).

### Magnetic resonance imaging

#### General

All MRI data were acquired at the F.M. Kirby Research Center for Functional Brain Imaging, using a whole-body 3T Philips Achieva System and a 32-channel receive-only head coil (Philips Healthcare, Best, The Netherlands). T_1_-, T_2_-, and T_2_-FLAIR scans were acquired for each participant and were assessed by a board-certified radiologist to confirm the absence of structural abnormalities or other incidental findings.

#### Resting state functional MRI (rs-fMRI)

All fMRI data were collected using a whole-brain ${\text{T}}_{2}^{*}$-weighted gradient-echo, echo planar imaging (GE-EPI) pulse sequence: TR = 2000 ms; TE = 30 ms; Flip Angle = 70°; SENSE Factor (AP/RL) = 2.0 (1.0/2.0); FOV (AP × FH × RL) = 200 mm × 104.5 mm × 180 mm; Number of Transverse Slices = 35 (ascending acquisition with 0.50 mm inter-slice gap); Spatial Resolution = 2.50 mm × 2.50 mm × 2.50 mm. With these parameters, each participant completed a 7.4-min resting state fMRI scan (i.e., 6 unused “steady-state” volumes followed by 216 rs-fMRI volumes) while staring at a white central fixation cue positioned centrally on a black background. The fixation cue was displayed on a rear-projection screen mounted in the distal bore of the scanner and was visible to participants via a mirror mounted directly on the head coil.

All images were spatially preprocessed in Matlab (The MathWorks Inc., Natick, MA), using SPM8 (http://www.fil.ion.ucl.ac.uk/spm/software/spm8/; Wellcome Trust Centre for Neuroimaging, London, UK), and were subsequently temporally preprocessed and analyzed using the Conn Toolbox (http://www.nitrc.org/projects/conn, Massachusetts Institute of Technology, Cambridge, MA) for functional connectivity analysis (Whitfield-Gabrieli and Nieto-Castanon, 2012). Spatial preprocessing of the rs-fMRI data included: (1) slice-time correction, (2) realignment to the mean T_2_*-weighted image, (3) coregistration to the high-resolution T_1_-weighted image, (4) spatial normalization to the ICBM152 template (Mazziotta et al., 1995) via unified segmentation (Ashburner and Friston, 2005), and (5) three-dimensional smoothing with a 6-mm full-width half-maximum (FWHM) Gaussian kernel.

Previous studies have highlighted the importance of taking additional measures to correct for participant motion (Power et al., 2012; Satterthwaite et al., 2012; Van Dijk et al., 2012) and physiological noise (Shmueli et al., 2007; Chang and Glover, 2009) in functional connectivity studies, since these factors can induce spurious correlations between brain regions, particularly among the low frequency time-series of interest in rs-fMRI experiments. Therefore, additional temporal preprocessing steps were performed in the Conn Toolbox to regress out the effects of participant motion (i.e., the six realignment parameters from rigid-body registration) and physiological motion (i.e., the time-courses of eroded white matter and cerebrospinal fluid masks using the CompCor method; Behzadi et al., 2007). The Artifact Detection Tool (http://www.nitrc.org/projects/artifact_detect/, Massachusetts Institute of Technology, Cambridge, MA) was then used to identify and regress out specific time-points with scan-to-scan intensity changes of *z* > 3.0, translational motion > 0.50 mm in any direction, and/or rotational motion > 0.05° in any plane. The rs-fMRI data were then temporally band-pass filtered (0.01–0.08 Hz) to isolate the low frequency fluctuations of interest (Biswal et al., 1995).

**Figure 2:**
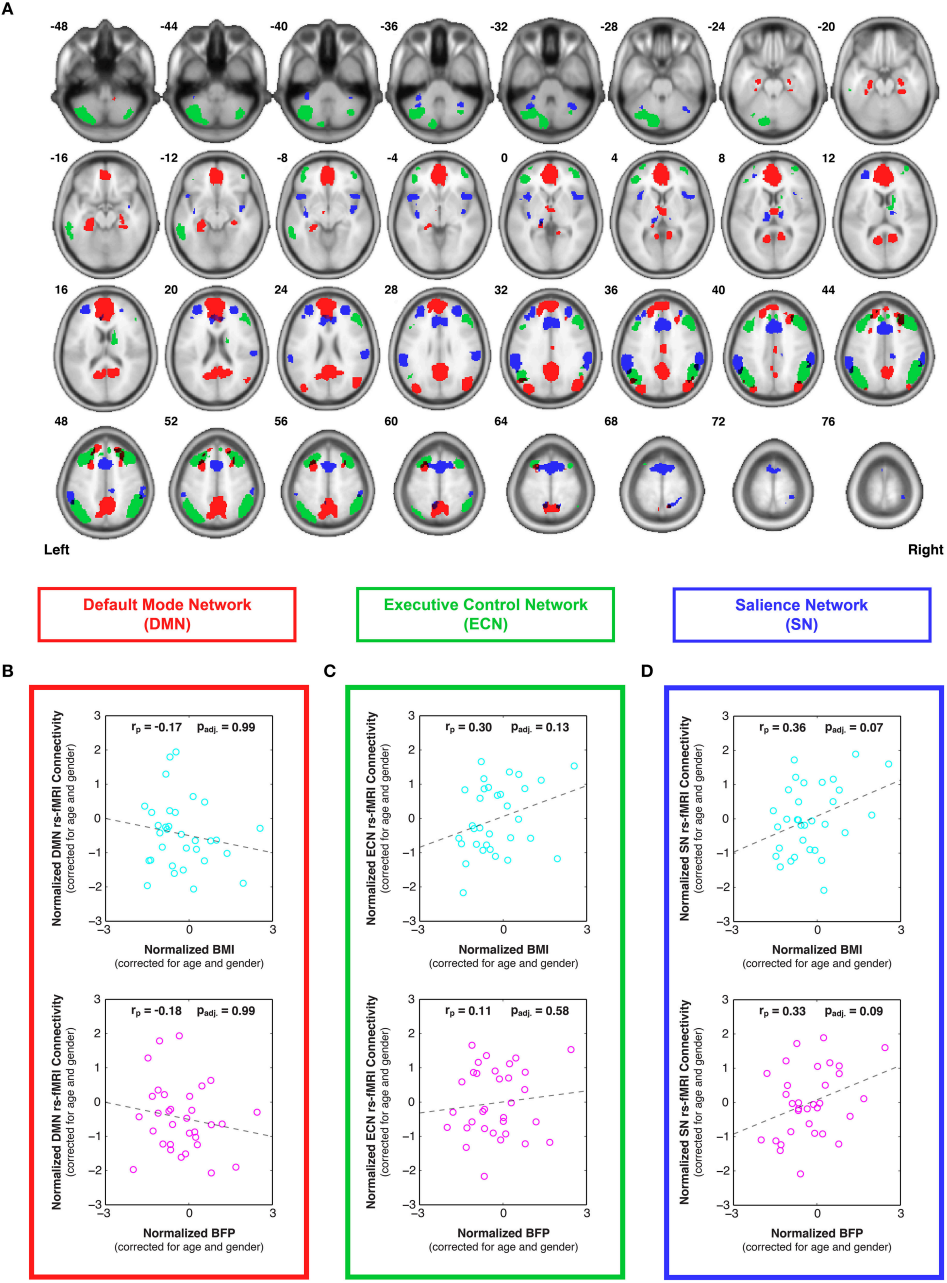
**Individual differences in resting state functional connectivity are correlated with body composition (i.e., BMI and/or BFP), corrected for age and gender. (A)** Previously defined regions of interest were taken from an anatomical atlas (Shirer et al., 2012) of three functionally distinct brain networks, including: all 19 nodes of the default mode network (DMN; red), all 12 nodes of the executive control network (ECN; green), and all 19 nodes of the salience network (SN; blue). After calculating average within-network connectivity values for each participant, partial correlations (corrected for age and gender) were performed between body composition data (BMI or BFP) and: **(B)** DMN connectivity, **(C)** ECN connectivity, and **(D)** SN connectivity. Neither measure of body composition was correlated with resting state DMN connectivity (panel **B**) or ECN connectivity (panel **C**) after correcting for multiple comparisons. However, both BMI and BFP were associated with marginally significant increases in SN connectivity (panel **D**; *p*_adj._ = 0.07 and *p*_adj._ = 0.09, respectively). All brain images are displayed in neurological orientation, and are in normalized MNI space.

After spatial and temporal preprocessing, data from each participant were subjected to a first-level region of interest (ROI) analysis to determine the bivariate correlations between each ROI-ROI pair within each network. All ROIs were defined *a priori* from an atlas of functionally connected brain networks (http://findlab.stanford.edu/functional_ROIs, Stanford University, Palo Alto, CA) (Shirer et al., 2012). The DMN was comprised of all 19 ROIs from the “dorsal” and “ventral” DMN, the ECN was comprised of all 12 ROIs from the “left” and “right” ECN, and the SN was comprised of all 19 ROIs from the “anterior” and “posterior” SN (Figure 2A). Following the first-level analysis, the Fisher-transformed connectivity values were averaged across all ROI-ROI combinations within each network to create average (within network) DMN, ECN, and SN connectivity scores for each participant. Partial correlation plots were then constructed to compare individual differences in functional connectivity within each network to BMI and BFP, with each axis corrected for age and gender (Figures 2B–D).

One potential consequence of evaluating three intrinsic brain networks is the overall inflation of type I errors due to multiple comparisons. To address this issue, non-parametric permutation tests (Nichols and Holmes, 2001) were performed in Matlab using the *mult_comp_perm_corr* function with 100,000 permutations and one-tailed *t*-tests (Groppe et al., 2011a,b). Since our null hypothesis was that no relationships existed between body composition and functional connectivity, the residuals of the BMI or BFP values (corrected for age and gender effects) were randomly permuted relative to the residuals of the functional connectivity measures (corrected for age and gender effects) across the DNM, ECN, and SN networks, and the distribution of the *maximum statistic* under the null hypothesis was then empirically determined by fitting the model after each permutation. This *maximum statistic* approach was used to determine adjusted *p*-values (*p*_adj._) for each network, which systematically limited the family-wise type I error rate to *p* < 0.05, while maximizing the statistical power in the event that different variables across the family of tests were inherently correlated (Groppe et al., 2011a,b). The directional hypotheses (i.e., one-tailed *t*-tests) were based on previously reported positive relationships between BMI and brain connectivity at the global level (Olde Dubbelink et al., 2008), as well as in the DMN (Kullmann et al., 2012) and regions of the SN (García-García et al., 2013).

#### High-resolution, T_1_-weighted voxel-based morphometry (VBM)

High-resolution, T_1_-weighted anatomical images were acquired with a 3D magnetization-prepared, rapid gradient echo (MP-RAGE) pulse sequence: TR = 7.93 ms; TE = 3.66 ms; Flip Angle = 8.00°; SENSE Factor (AP/RL/FH) = 2.4 (2.0/1.0/1.2); FOV (AP × FH × RL) = 212 mm × 150 mm × 172 mm; Spatial Resolution = 1.00 mm × 1.00 mm × 1.00 mm; Scan Duration = 4 min and 26 s.

Morphometric analyses were performed with SPM8, using the VBM8 Toolbox (http://dbm.neuro.uni-jena.de/vbm/, Friedrich Schiller University of Jena, Jena, Germany) to assess regional differences in gray matter (GM) and white matter (WM) volumes that were associated with body composition (i.e., BMI and BFP). For each participant, the T_1_-weighted images were partitioned into separate GM and WM tissue classes by means of unified segmentation (Ashburner and Friston, 2005). These classes were then normalized to a custom-made, sample-based template using the high-dimensional, non-linear DARTEL warping algorithm (Ashburner, 2007; Ashburner and Friston, 2009). After performing image modulation (to compensate for regional volume changes imparted during the previous non-linear spatial normalization step), GM and WM images were smoothed with a three-dimensional 5.00 mm FWHM Gaussian kernel. The preprocessed images were then submitted to a second-level general linear model analysis (Friston et al., 1995) to assess voxel-wise correlations between tissue volumes (i.e., GM and WM) and either BMI or BFP.

**Figure 3:**
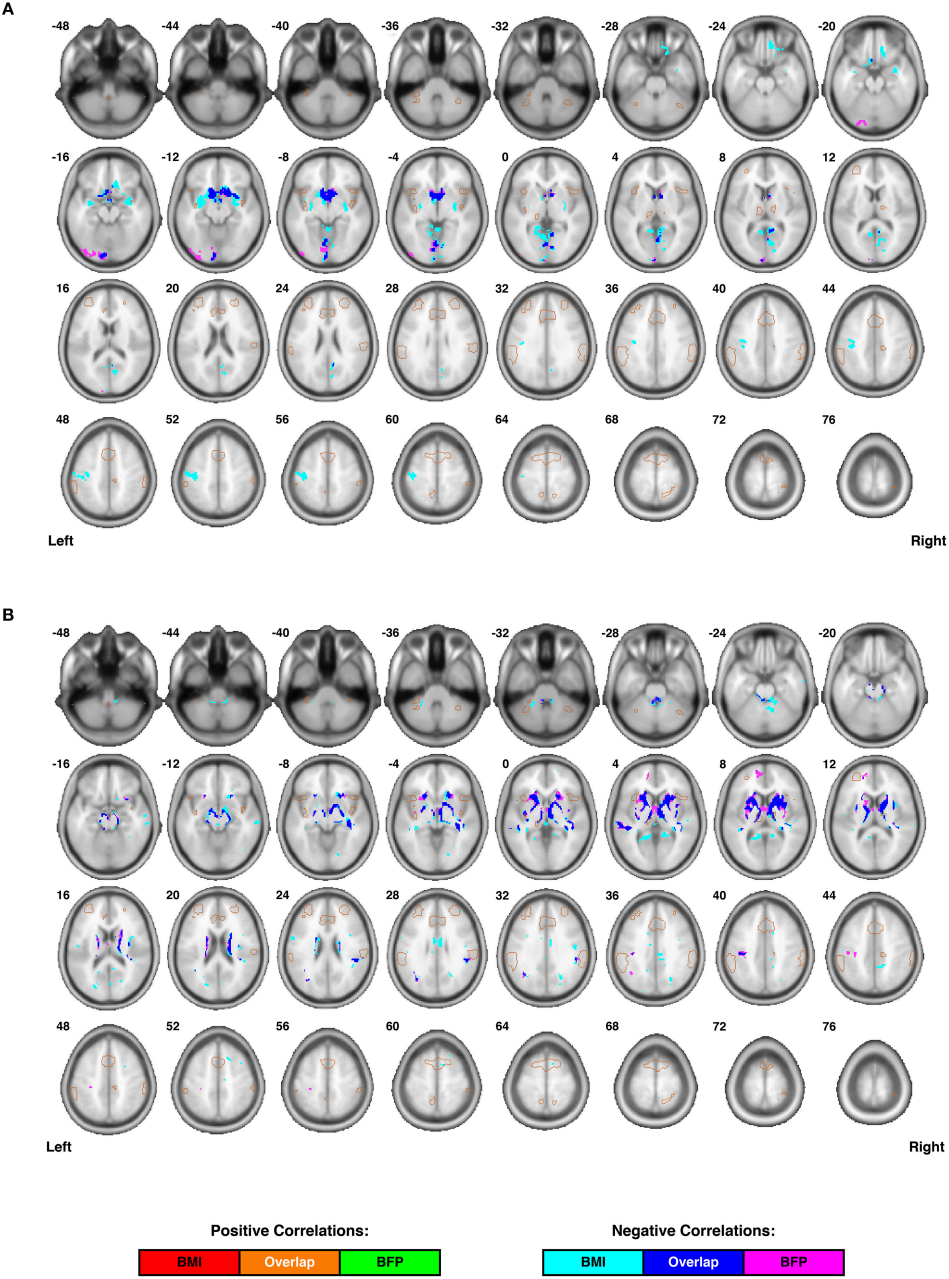
**Individual differences in regional cortical volumes are correlated with body composition (i.e., BMI and/or BFP), corrected for age and gender. (A)** Gray matter volumes were negatively correlated with BMI in three clusters and negatively correlated with BFP in two clusters (see Table 1 for details regarding significance, size, and location of each cluster). No gray matter structures demonstrated significant positive correlations with either BMI or BFP. **(B)** White matter volumes were negatively correlated with BMI in six clusters and negatively correlated with BFP in seven clusters (see Table 1 for details regarding significance, size, and location of each cluster). No white matter structures demonstrated significant positive correlations with either BMI or BFP. All brain images are displayed in neurological orientation, and are in normalized MNI space. Regions of the salience network (SN) are indicated by light brown outlines for reference.

To control for individual differences in age, gender, and total intracranial volume (TICV) in these correlations: (1) age and gender for each participant were included in the second-level analyses as regressors of no interest, and (2) global normalization (“proportional scaling”) was used to correct for individual differences in TICV (i.e., the cumulative volume of gray matter, white matter, and cerebrospinal fluid). Finally, second-level statistical analyses were masked with single-subject DARTEL segmentations to include only the tissue of interest (i.e., GM or WM, respectively), and all cluster-level significance values were corrected for multiple comparisons using the family-wise error (FWE) and false discovery rate (FDR) approaches embedded in SPM8 (Figure 3 and Table 1).

**Table 1:** **Cluster-level statistics for VBM results (i.e., regional gray and white matter volumes that were correlated with BMI or BFP, corrected for age and gender)**.

**Tissue category (gray or white matter) vs. body composition metric (BMI or BFP)**	**Cluster number**	**FWE-corrected *p*-value**	**FDR-corrected *p*-value**	**Number of voxels**	**MNI coordinates (x, y, z)**	**Brain region**
Gray matter volume vs. BMI (positive)	No significant clusters	N/A	N/A	N/A	N/A	N/A
Gray matter volume vs. BMI (negative)	Cluster #1	0.000	0.000	4077	−6, 12, −15	Left olfactory lobule
					32, −6, −15	Right amygdala
					−6, 3, −8	Left putamen
	Cluster #2	0.000	0.000	2751	9, −55, 22	Right precuneus
					9, −78, −2	Right lingual gyrus
					−9, −58, −2	Left lingual gyrus
	Cluster #3	0.006	0.003	1023	−42, −21, 60	Left precentral gyrus
					−36, −15, 39	Left postcentral gyrus
					−51, −15, 54	Left postcentral gyrus
Gray matter volume vs. BFP (positive)	No significant clusters	N/A	N/A	N/A	N/A	N/A
Gray matter volume vs. BFP (negative)	Cluster #1	0.000	0.000	1932	−6, 12, −12	Left caudate
					3, 3, −6	Right caudate
					6, 14, −9	Right olfactory
	Cluster #2	0.000	0.000	1754	−8, −90, −14	Left calcarine
					−44, −87, −8	Left inferior occipital
					−6, −99, 9	Left superior occipital
White matter volume vs. BMI (positive)	No significant clusters	N/A	N/A	N/A	N/A	N/A
White matter volume vs. BMI (negative)	Cluster #1	0.000	0.000	5367	8, −7, −5	Right cerebral peduncle
					46, −36, 27	Right supramarginal white matter
					8, −1, 1	Right internal capsule
	Cluster #2	0.000	0.000	2397	−30, −1, 9	Left external capsule
					−20, 17, −0	Left putamen
					−15, −15, 10	Left thalamus
	Cluster #3	0.004	0.004	311	−44, −33, 3	Left superior temporal gyrus
					−62, −25, 4	Left superior temporal gyrus
	Cluster #4	0.007	0.005	283	−15, −49, 7	Left cingulum (hippocampus)
					−15, −55, 15	Left cingulate gyrus
					−26, −52, 4	Left lingual gyrus
	Cluster #5	0.030	0.019	210	−6, −1, 28	Left body of the corpus callosum
					8, −1, 28	Right body of the corpus callosum
					−9, −9, 34	Left body of the corpus callosum
	Cluster #6	0.075	0.040	169	−34, −57, 33	Left angular white matter
					−38, −51, 21	Left middle temporal white matter
					−30, −46, 33	Left superior longitudinal fasciculus
White matter volume vs. BFP (positive)	No significant clusters	N/A	N/A	N/A	N/A	N/A
White matter volume vs. BFP (negative)	Cluster #1	0.000	0.000	6710	21, 15, 6	Right anterior limb of internal capsule
					4, −3, 0	Right thalamus
					32, 3, 9	Right external capsule
	Cluster #2	0.000	0.000	671	50, −34, 28	Right supramarginal white matter
					36, −30, −3	Right inferior longitidinal fasciculus/fronto−occipital fasciculus
					30, −34, 9	Right fornix cres/striata terminalis
	Cluster #3	0.035	0.026	294	−58, −27, 3	Left superior temporal gyrus
					−45, −31, 3	Left superior temporal gyrus
					−38, −36, 7	Left superior temporal white matter
	Cluster #4	0.091	0.046	236	−33, −49, 33	Left angular white matter/superior longitudinal fasciculus
					−36, −57, 31	Left angular gyrus
					−33, −69, 25	Left middle occipital white matter
	Cluster #5	0.099	0.046	231	−39, −24, 39	Left postcentral gyrus
					−30, −27, 46	Left superior longitudinal fasciculus
					−28, −31, 55	Left postcentral gyrus
	Cluster #6	0.153	0.054	205	3, −37, −30	Right medial lemniscus
					−4, −42, −29	Left superior cerebellar peduncle
					−8, −34, −18	Left superior cerebellar peduncle
	Cluster #7	0.158	0.054	203	−18, 50, 12	Left superior frontal white matter
					−10, 56, 7	Left superior frontal gyrus
					−15, 48, 4	Left superior frontal gyrus

*Corrected p-values represent the probability that each cluster has been falsely identified as significant after correcting for multiple comparisons with family-wise error (FWE) or false discovery rate (FDR) approaches. MNI coordinates and the approximate brain regions—identified using the TD-ICBM Human Atlas in the SPM8 WFU PickAtlas Toolbox (http://fmri.wfubmc.edu/software/PickAtlas; Wake Forest University, Winston-Salem, NC)—represent the local maxima within each cluster (>8 mm apart). Please note that corresponding data are shown in Figure 3*.

#### Diffusion tensor imaging (DTI)

Diffusion-weighted images were acquired with a previously reported single-shot spin echo, echo-planar imaging (SE-EPI) pulse sequence (Farrell et al., 2007): 30 diffusion-encoded images (*b* = 700 s/mm^2^); 5 reference images (*b* = 0 s/mm^2^); TR = 6904 ms; TE = 69 ms; Flip Angle = 90°; SENSE Factor (AP/RL) = 2.5 (2.5/1.0); FOV (AP × FH × RL) = 212 mm × 154 mm × 212 mm; Number of Transverse Slices = 70 (no inter-slice gap); Spatial Resolution (Acquired) = 2.20 mm × 2.20 mm × 2.20 mm; Spatial Resolution (Resampled) = 0.83 mm × 0.83 mm × 2.20 mm; Scan Duration = 4 min and 16 s.

Diffusion weighted data were preprocessed with CATNAP (Coregistration, Adjustment, and Tensor-solving, a Nicely Automated Program; http://iacl.ece.jhu.edu/~bennett/catnap/, JHU School of Medicine, Baltimore, Maryland, USA) to motion-correct and coregister each participant's diffusion-weighed images to the reference images (i.e., the mean *b* = 0 s/mm^2^ image) using 12-parameter (affine) registration, which also corrects for eddy current distortions. Within CATNAP, the gradient directions for each diffusion-weighted image were automatically extracted from the scanner settings (i.e., slice angulation, orientation, and order) and reoriented to account for the previous coregistration and motion-correction step before calculating the six tensor images (d_xx_, d_yy_, d_zz_, d_xy_, d_xz_, d_yz_) (Landman et al., 2007). The reference image and all six tensor images were then skull stripped using a two-step procedure, in which: (1) participant-specific brain masks were generated using the New Segment Tool in SPM8, and (2) these masks were manually refined using the ROIEditor Toolbox in MRIStudio (https://www.mristudio.org/, JHU School of Medicine, Baltimore, Maryland, USA).

The motion-corrected, coregistered, and skull-stripped images for each participant were then normalized to the JHU, Montreal Neurological Institute (MNI) coordinate, single-subject, mean *b* = 0 s/mm^2^, skull-stripped (i.e., “JHU_MNI_SS_b0_ss”) template (Mori et al., 2008). This procedure was implemented using the DiffeoMap Toolbox in MRIStudio to carry out a 12-parameter (affine) transformation, followed by high-dimensional, non-linear warping via the Large Deformation Diffeomorphic Metric Mapping (LDDMM) algorithm (Beg et al., 2005). To allow increasingly elastic deformations, which have been shown to produce optimal image registration (Ceritoglu et al., 2009), a three-stage LDDMM analysis was performed with cascading deformation elasticity (i.e., “alpha value”) inputs of 0.01, 0.005, and 0.002.

**Figure 4:**
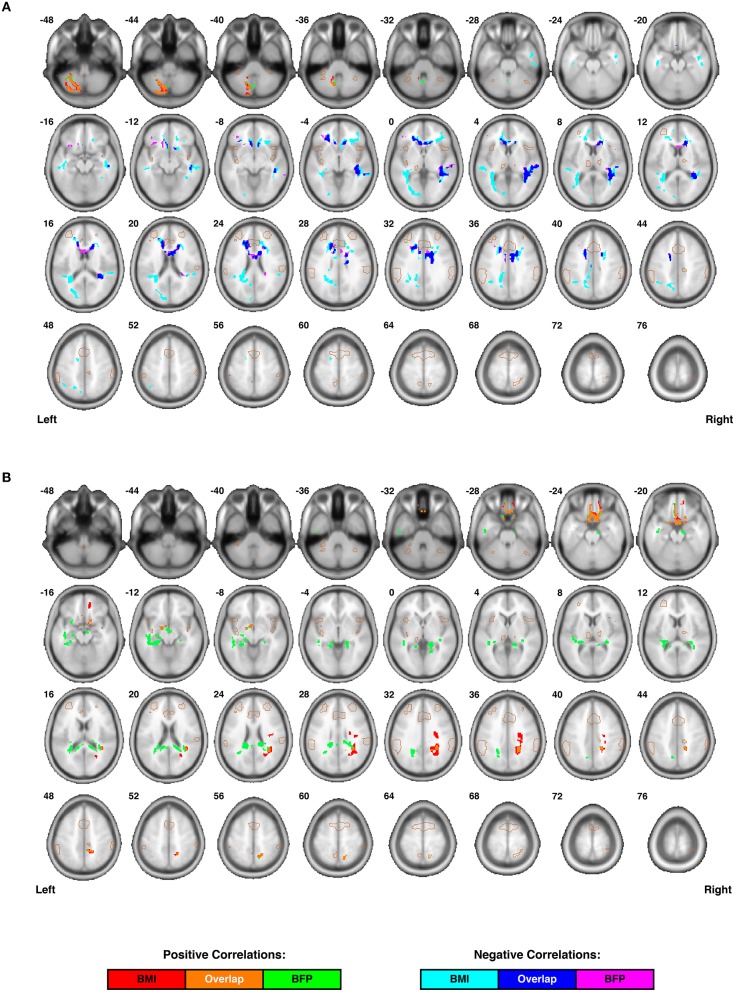
**Individual differences in regional white matter microstructure are correlated with body composition (i.e., BMI and/or BFP), corrected for age and gender. (A)** Fractional anisotropy (FA) values were negatively correlated with BMI in three clusters, negatively correlated with BFP in two clusters, and positively correlated with both BMI and BFP in one cluster (see Table 2 for details regarding significance, size, and location of each cluster). **(B)** Mean diffusivity (MD) values were positively correlated with BMI in three clusters and negatively correlated with BFP in two clusters (see Table 2 for details regarding significance, size, and location of each cluster). No white matter structures demonstrated significant negative correlations with either BMI or BFP. All brain images are displayed in neurological orientation, and are in normalized MNI space. Regions of the salience network (SN) are indicated by light brown outlines for reference.

After applying the overall Kimap (i.e., affine plus LDDMM non-linear transformations) to each participant's tensor images, the data were loaded into the DTIStudio Toolbox (Jiang et al., 2006) within MRIStudio to generate normalized fractional anisotropy (FA) and mean diffusivity (MD) maps. After smoothing the normalized FA and MD images with a three-dimensional 6.00 mm FWHM Gaussian kernel, a second-level general linear model analysis was performed in SPM8 to assess individual differences in white matter microstructure that correlated with BMI or BFP, corrected for age and gender (similar to the aforementioned VBM analysis). To restrict the statistical analyses of the FA and MD maps to white matter regions, a ROI analysis was performed using a single-subject white matter mask (generated during the previous VBM analysis). All results are shown and reported as cluster-level significance values, corrected for multiple comparisons using the FWE and FDR approaches embedded in SPM8 (Figure 4 and Table 2).

**Table 2:** **Cluster-level statistics for DTI results (i.e., regional white matter fractional anisotropy (FA) and mean diffusivity (MD) values that were correlated with BMI or BFP, corrected for age and gender)**.

**Microstructural measure (FA or MD) vs. body composition metric (BMI or BFP)**	**Cluster number**	**FWE-corrected *p*-Value**	**FDR-corrected *p*-Value**	**Number of voxels**	**MNI coordinates (x, y, z)**	**Brain region**
Fractional anisotropy vs. BMI (positive)	Cluster #1	0.046	0.048	1124	−10, −84, −46	Left cerebellum
					−22, −64, −46	Left cerebellum
					−24, −80, −48	Left cerebellum
Fractional anisotropy vs. BMI (negative)	Cluster #1	0.000	0.006	2874	28, 28, −6	Right lateral fronto-orbital white matter
					−16, 28, 28	Left anterior corona radiata
					−22, 34, −2	Left anterior corona radiata
	Cluster #2	0.003	0.006	2005	−34, −60, 4	Left posterior thalamic radiation
					−42, −42, 20	Left superior temporal gyrus
					−54, −26, 6	Left superior temporal white matter
	Cluster #3	0.031	0.006	1444	50, −26, 6	Right superior temporal white matter
					46, −32, −8	Right inferior longitidinal fasciculus/inferior fronto-occipital fasciculus
					42, −42, 0	Right middle temporal white matter
Fractional anisotropy vs. BFP (positive)	Cluster #1	0.554	0.049	824	−22, −64, −46	Left middle cerebellar peduncle
					−10, −84, −46	Left cerebellum
					−10, −64, −36	Left cerebellum
Fractional anisotropy vs. BFP (negative)	Cluster #1	0.001	0.001	2014	−18, 22, 32	Left superior corona radiata
					−22, 34, −2	Left anterior corona radiata
					20, −8, 30	Right superior corona radiata
	Cluster #2	0.123	0.049	935	38, −32, 2	Right retrolenticular part of internal capsule
					34, −42, 12	Right posterior thalamic radiation
					50, −26, 6	Right superior temporal white matter
Mean diffusivity vs. BMI (positive)	Cluster #1	0.551	0.079	941	24, −58, 26	Right precuneus white matter
					20, −42, 38	Right cingulum white matter
					26, −60, 18	Right cuneus
	Cluster #2	0.881	0.079	749	−6, 12, −26	Left frontal white matter
					10, 6, −22	Right frontal white matter
					−2, 22, −20	Left gyrus rectus (straight gyrus)
Mean diffusivity vs. BMI (negative)	No significant clusters	N/A	N/A	N/A	N/A	N/A
Mean diffusivity vs. BFP (positive)	Cluster #1	0.103	0.031	1372	−22, −38, 22	Left tapetum of corpus callosum
					−26, −40, −8	Left para-hippocampal gyrus
					−14, −42, 18	Left splenium of corpus callosum
	Cluster #2	0.477	0.031	982	20, −38, 0	Right cingulum (near hippocampus)
					20, −42, 40	Right superior parietal white matter
					12, −38, 16	Right splenium of corpus callosum
	Cluster #3	0.811	0.031	794	−8, 14, −26	Left frontal white matter
					2, 28, −32	Right gyrus rectus (straight gyrus)
					14, 20, −26	Right middle fronto-orbital gyrus
Mean diffusivity vs. BFP (negative)	No significant clusters	N/A	N/A	N/A	N/A	N/A

*Corrected p-values represent the probability that each cluster has been falsely identified as significant after correcting for multiple comparisons with family-wise error (FWE) or false discovery rate (FDR) approaches. MNI coordinates and the approximate brain regions—identified using the TD-ICBM Human Atlas in the SPM8 WFU PickAtlas Toolbox (http://fmri.wfubmc.edu/software/PickAtlas; Wake Forest University, Winston-Salem, NC)—represent the local maxima within each cluster (>8 mm apart). Please note that corresponding data are shown in Figure 4*.

#### White matter analyses of the DMN, ECN, and SN

In addition to the whole-brain voxel-wise analyses described above, relationships between body composition and individual differences in white matter regions underlying the DMN, ECN, and SN were also specifically examined. ROI-based analyses were performed on each subject's spatially-normalized VBM and DTI data in order to extract white matter volume, FA, and MD measures from fMRI-guided DTI atlases of the dorsal and ventral DMN, left and right ECN, and anterior and posterior SN, which were recently reported in a separate paper by our group (Figley et al., 2015). Since the atlases for each network were originally defined as two ROIs (e.g., dorsal DMN and ventral DMN), quantitative white matter imaging values were extracted from both ROIs in order to estimate average tissue volume, FA, and MD values within each white matter network for each subject.

**Figure 5:**
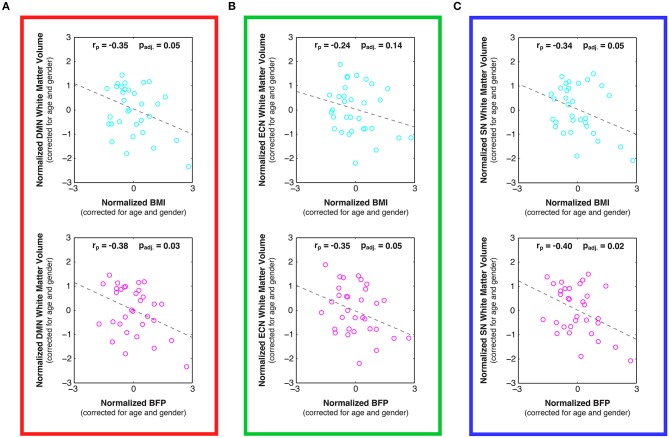
**Linear relationships between body composition (i.e., BMI and BFP) and white matter volume within previously-defined stereotaxic atlases of (A) default mode network (DMN), (B) executive control network (ECN), and (C) salience network (SN) white matter regions (Figley et al., 2015), corrected for age and gender**. Higher BMI and BFP were associated with statistically significant decreases in white matter volume in both the DMN (panel **A**; *p*_adj._ = 0.05 and *p*_adj._ = 0.03, respectively) and SN (panel **C**; *p*_adj._ = 0.05 and *p*_adj._ = 0.02, respectively); and although the relationship between BMI and ECN white matter volume was not significant (panel **B**; *p*_adj._ = 0.14), BFP was associated with significantly decreased ECN white matter volume (panel **B**; *p*_adj._ = 0.05).

**Figure 6:**
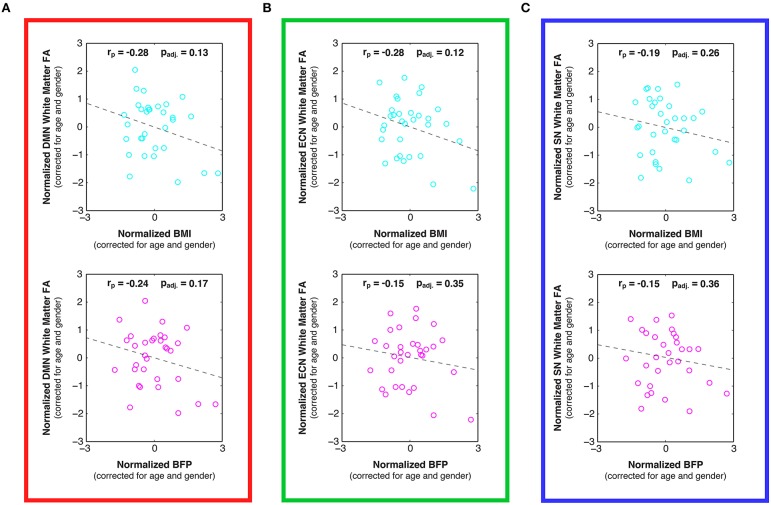
**Linear relationships between body composition (i.e., BMI and BFP) and white matter fractional anisotropy (FA) within previously-defined stereotaxic atlases of (A) default mode network (DMN), (B) executive control network (ECN), and (C) salience network (SN) white matter regions (Figley et al., 2015), corrected for age and gender**. Although not statistically significant after correcting for multiple comparisons, trends were observed between increased BMI and decreased FA in the DMN (panel **A**; *p*_adj._ = 0.13) and ECN (panel **B**; *p*_adj._ = 0.12), as well as between increased BFP and decreased FA in the DMN (panel **A**; *p*_adj._ = 0.17).

**Figure 7:**
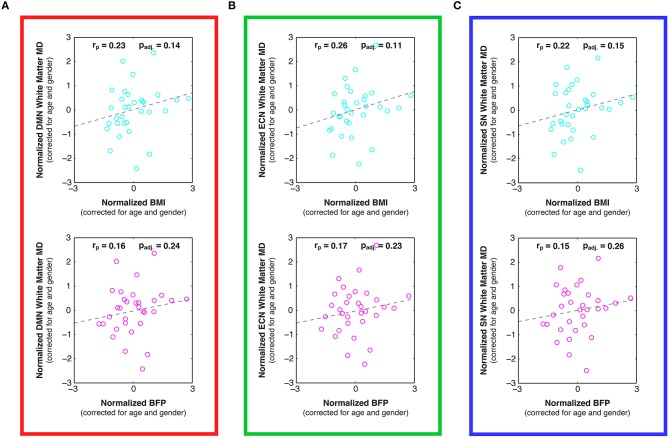
**Linear relationships between body composition (i.e., BMI and BFP) and white matter mean diffusivity (MD) within previously-defined stereotaxic atlases of (A) default mode network (DMN), (B) executive control network (ECN), and (C) salience network (SN) white matter regions (Figley et al., 2015), corrected for age and gender**. Although not statistically significant after correcting for multiple comparisons, trends were observed between increased BMI and decreased MD in the DMN (panel **A**; *p*_adj._ = 0.14), ECN (panel **B**; *p*_adj._ = 0.11), and SN (panel **C**; *p*_adj._ = 0.15).

White matter volume measurements (Figure 5) were extracted from the spatially normalized and modulated white matter images used for the VBM analyses described above, whereas FA (Figure 6) and MD (Figure 7) values were extracted directly from the corresponding high-dimensional, non-linear normalized images used for the previously described voxel-wise analyses. Linear regressions were then performed between each white matter measure (i.e., volume, FA, or MD) and each body composition measure (i.e., BMI or BFP) across all 32 subjects, with both axes corrected for age and gender. As in the rs-fMRI connectivity analyses, non-parametric permutation testing was performed for each imaging biomarker vs. body composition relationship (using 100,000 permutations, one-tailed *t*-tests, and the maximum statistic approach) in order to correct for multiple comparisons and to limit the estimated FWE to *p* < 0.05 across all three networks.

## Results

### Body composition

Although the mean age (28.7 ± 9.7 vs. 30.9 ± 11.5 years; two-tailed *p* = 0.55) was not significantly different between males and females in our sample population, there were marginally significant sex differences in BMI (26.2 ± 4.4 vs. 23.5 ± 4.2 kg/m^2^; two-tailed *p* = 0.09), and BFP (20.4 ± 10.0 vs. 26.8 ± 9.1 percent; two-tailed *p* = 0.07), such that males appeared to have higher average BMI and lower average BFP. Therefore, although data from both sexes were combined in subsequent functional and structural MRI analyses to increase statistical power, partial correlations were used to regress out individual differences in age and/or sex. Importantly, participants in our sample spanned a wide range of BMI and BFP values (Figure 1 and Figure S1)—including categorically underweight, normal weight, overweight, and obese individuals—which enabled individual differences in body composition to be correlated with functional connectivity, gray and white matter morphology, and white matter microstructure (described below).

### Correlations with rs-fMRI functional connectivity

Resting state fMRI (rs-fMRI) data were used to evaluate functional connectivity strength within previously reported brain networks, including the DMN, ECN, and SN. The specific regions of interest for each network (Figure 2A) were taken from a pre-existing functional connectivity atlas (Shirer et al., 2012), and the bivariate correlations were averaged between all ROI pairs within each network to determine overall network connectivity strengths for each participant. After correcting for multiple comparisons across networks, the partial correlation plots of network connectivity vs. body composition (previously corrected for individual differences in age and sex) showed no significant relationships between DMN connectivity and either BMI or BFP (Figure 2B; *p*_adj._ = 0.99 and *p*_adj._ = 0.99, respectively)^2^, and no significant relationships between ECN connectivity and either BMI or BFP (Figure 2C; *p*_adj._ = 0.13 and *p*_adj._ = 0.58, respectively). However, SN connectivity showed marginally significant positive correlations with both BMI and BFP, even after correcting for multiple comparisons (Figure 2D; *p*_adj._ = 0.07 and *p*_adj._ = 0.09, respectively), such that higher BMI and BFP were associated with greater SN connectivity.

### Correlations with regional gray and white matter volumes

Voxel-based morphometry (VBM) was carried out on participants' high-resolution T_1_-weighted images to identify regional volumetric changes related to body composition. Gray and white matter volumes were analyzed separately (based on the segmented tissue masks) and were corrected for the effects of age, gender, and TICV in each analysis. Cluster-level analyses suggested that gray matter volumes were negatively correlated with both BMI and BFP, primarily in large portions of the bilateral dorsal striatum and medial occipital cortex (Figure 3A and Table 1).

White matter volumes in several regions were also negatively correlated with BMI and BFP (Figure 3B and Table 1). The most prominent white matter reductions were observed in regions surrounding the dorsal striatum (i.e., internal and external capsules), corpus callosum (CC), ILF, superior longitudinal fasciculus (SLF), and parts of the fronto-occipital fasciculus (FOF).

Interestingly, neither BMI nor BFP were associated with gray or white matter volume increases in any region, and many of the clusters exhibiting regional volume decreases appeared to be in close proximity to each other, as well as to several of the DMN, ECN, and SN ROIs (Figure 2A).

### Correlations with global gray and white matter volumes

In addition to analyzing regional gray and white matter volume changes, we assessed relationships between body composition (i.e., BMI and BFP) and global tissue volumes. The global GM or WM volume for each subject was determined by multiplying the number of voxels in each segmented tissue mask by the volume of each voxel (i.e., the spatial resolution). After correcting for age, gender, and TICV, we found positive correlations between body composition (i.e., BMI and BFP) and global gray matter volume (Figure S2A; *p* = 0.07 and *p* = 0.04, respectively), no correlations with global white matter volume (Figure S2B; *p* = 0.80 and *p* = 0.57, respectively), and marginally significant negative correlations with global CSF volume (Figure S2C; *p* = 0.08 and *p* = 0.07, respectively).

### Correlations with regional white matter microstructure

Relationships between body composition and white matter microstructure were assessed by calculating normalized, whole-brain fractional anisotropy (FA) and mean diffusivity (MD) maps from each subject's DTI data and then performing multiple linear regressions to identify regional (i.e., cluster-wise) correlations with either BMI or BFP (corrected for age and gender). These DTI measures were chosen because they provide complementary information. For example, FA is a highly sensitive measure of tissue microstructure, but may lack mechanistic specificity; on the other hand, MD is generally thought to measure specific changes in edema (swelling), cellularity, and overall membrane density, but may be less sensitive than FA (Beaulieu, 2002; Alexander et al., 2011).

Increased FA (i.e., greater microstructural integrity) was correlated with both higher BMI and BFP in a single cluster located in the left cerebellum. However, elevated BMI and BFP were also associated with significantly decreased FA (i.e., lower microstructural integrity) in several regions (Figure 4A and Table 2), including the white matter surrounding the dorsal striatum (including regions of the corona radiata and internal capsule), thalamic radiation, genu of the corpus callosum, orbitofrontal white matter, temporal white matter, and regions of the ILF.

Neither BMI nor BFP were associated with decreased MD (i.e., greater microstructural integrity), but body composition was associated with increased MD (i.e., lower microstructural integrity) in several regions (Figure 4B and Table 2). These areas included the: (1) olfactory bulb, gyrus rectus, and orbitofrontal cortex (which were also observed to have decreased gray matter volume), (2) parahippocampal gyri (which were also observed to have decreased gray and white matter volumes), (3) cingulum (particularly areas close to the hippocampus), (4) splenium of the corpus callosum, (5) bilateral cuneus/precuneus, and (6) right superior temporal white matter. Much like the regional volumetric changes described earlier, many of the white matter regions showing decreased FA and increased MD were in close proximity to one another, as well as various DMN, ECN, and SN ROIs (Figure 2A).

### Correlations with white matter volume and microstructure in the DMN, ECN, and SN

In addition to the voxel-wise white matter analyses reported above, stereotaxic white matter atlases of the DMN, ECN, and SN (Figley et al., 2015) were also used to perform ROI-based analyses for each of the networks examined with rs-fMRI. Despite the fact that neither body composition measure was related to individual differences in global white matter volumes (see above), white matter volumes within all three functionally-defined networks were negatively correlated with BMI and BFP, and all but one of these associations was statistically significant, even after correcting for multiple comparisons across networks (Figure 5; *p*_adj._ < 0.05, except for BMI vs. ECN white matter volume).

Examining the DTI data throughout these networks revealed that higher BMI and BFP were also associated with trending decreases in FA (Figure 6) and trending increases in MD (Figure 7), which are both indicative of reduced tissue microstructure. However, none of the trends with tissue microstructure remained statistically significant after correcting for multiple comparisons.

### Possible relationships to functional connectivity throughout the basal ganglia network

After observing widespread volumetric and microstructural changes throughout the dorsal striatum (Figure 3 and Figure 4), we reanalyzed the rs-fMRI data to evaluate possible relationships between body composition (i.e., BMI and BFP) and intrinsic basal ganglia network (BGN) connectivity (i.e., in addition to the original DMN, ECN, and SN analyses). Although this analysis had certain limitations that went beyond the scope of this study and could be improved upon in future studies (see Supplementary Information), it did not reveal any significant correlations between intrinsic BGN connectivity and BMI or BFP after correcting for individual differences in age and gender (Figure S3; *p* = 0.93 and *p* = 0.90, respectively).

## Discussion

### Novelty of the current study

In addition to acquiring functional and structural neuroimaging data within the same sample population—which, to the best of our knowledge, has not been previously reported—our fundamental approach to assessing body composition differed from most previous neuroimaging studies in four key respects. First, although we calculated BMI for every participant (similar to most previous studies), we also measured BFP with bioelectric impedance to get an independent, and arguably better, estimate of body fat. Second, instead of binning participants' BMI or BFP data into categories (e.g., “lean” vs. “obese”) and performing categorical or group-wise analyses, all of our results were based on linear regressions of the raw body composition metrics, thereby taking advantage of even subtle individual differences. Third, all of our structural neuroimaging data were subjected to high-dimensional, non-linear warping (i.e., DARTEL or LDDMM) to facilitate whole-brain, voxel-wise analyses (with cluster-level statistics) to avoid *a priori* assumptions about specific brain regions of interest. And finally, we used previously published fMRI (Shirer et al., 2012) and DTI (Figley et al., 2015) atlases to assess resting state functional connectivity, white matter volume, and white matter microstructure throughout three brain networks associated with cognition, executive control, and salience processing (i.e., the DMN, ECN, and SN).

Overall, our results confirm that individual differences in BMI and BFP are associated with widespread differences in brain function and structure among otherwise healthy adults, independent of age and gender effects. Unlike previous studies, however, we found converging evidence across multiple structural and functional imaging modalities to identify a core set of brain regions and networks that: (1) appear to be related to differences in body composition, and (2) may partly explain previously reported cognitive and behavioral effects associated with obesity.

In particular, the current findings indicate that increased BMI and BFP are negatively associated with regional gray and white matter volumes (Figure 3) and white matter microstructure (Figure 4) in brain regions associated with value assessment, habit formation, and decision-making (e.g., the striatum and orbitofrontal cortex). Upon closer examination of the default mode, executive control, and salience networks, higher BMI and BFP were also associated with increased resting state functional connectivity throughout the SN (Figure 2) and with corresponding alterations in tissue morphology: particularly, reduced DMN, ECN, and SN white matter volumes (Figure 5). The concurrent finding that body composition was associated with significant volume decreases within these white matter networks, but had no apparent relation to global white matter volume (Figure S2), implies that higher BMI and BFP subjects had increased white matter volumes that were either dispersed or inconsistent in terms of their locations in other brain regions (Figure 3).

### Different measures of body composition

Despite an imperfect correlation between BMI and BFP in our sample (*r* = 0.70; *r*_*p*_ = 0.89 corrected for age and gender), most of the functional and structural correlates of body composition were apparent using either measure. Voxel-wise statistical maps generated with both body composition measures (Figures 3, 4) generally showed a high degree of spatial overlap, and the partial correlations with functional and structural connectivity throughout the DMN, ECN, and SN (Figures 2,5–7) were generally in good correspondence. Therefore, although results will almost certainly differ within certain demographics (e.g., among body-builders or other professional athletes), our results generally appear to validate previous imaging studies using BMI as a measure of body composition in “normal” sample populations.

### Our findings within the context of previous literature

Overall, our findings strongly support the notion that there are distinct neural correlates of body composition, which may be linked to both physical and mental health (c.f. Scott et al., 2008; Marcus and Wildes, 2009). In particular, our rs-fMRI results support and extend previous findings that elevated body fat is associated with increased functional connectivity in the SN (García-García et al., 2013). However, unlike the prior study—which reported significant connectivity differences in the putamen and only trending increases among insular and superior parietal regions (García-García et al., 2013)—our data suggest that intrinsic connectivity is significantly increased (on average) throughout the entire salience-processing network (Figure 2). These distributed functional changes fit with previous task-based fMRI studies in which tastes and/or pictures of high-calorie foods elicited distributed neural activity throughout many of the regions identified in our study—i.e., the dorsal striatum, as well as gustatory, limbic, and reward regions—and that these responses were disproportionately large in overweight vs. normal weight participants (Simmons et al., 2005; Porubská et al., 2006; Rothemund et al., 2007; Stoeckel et al., 2008) and reduced in anorexic patients (McFadden et al., 2014).

On the contrary, individual differences in BMI and BFP in our sample were not correlated with functional connectivity throughout the DMN, as previously reported (Kullmann et al., 2012), despite the slightly larger sample size and longer rs-fMRI scans employed in the current study. One possible explanation for the disparate finding is that partial correlations in the previous report regressed out effects of fasting insulin levels, as opposed to age and sex differences that were regressed in the current study. However, an even more likely explanation is that the previous study by Kullmann et al. calculated functional connectivity strengths of each region separately and then reported regions for which connectivity was significantly increased (e.g., the left precuneus) and or decreased (e.g., the right anterior cingulate cortex). Therefore, because BMI and BFP were correlated with *average* network connectivity strength in the current study (i.e., the mean of all ROI-ROI bivariate correlations within the network), positive correlations between some network nodes and negative correlations between others would have been averaged out in our analysis, providing a single measure of overall connectivity within the network. This approach was chosen in our study to drastically reduce the multiple comparisons problems that would have otherwise been significant had we chosen to examine all ROI-ROI combinations separately. Nonetheless, although we did not observe any significant relationships with resting state connectivity in the DMN as a whole, data from other studies—e.g., those showing greater activity in the posterior cingulate and left inferior parietal cortices among reduced-obese patients (i.e., individuals who were obese, but had recently undergone rapid and significant weight loss) to healthy normal-weight controls (Tregellas et al., 2011) or those demonstrating that exercise-related body fat losses are met with reduced connectivity of the precuneus (McFadden et al., 2013)—suggest that body composition may be related to activity and functional connectivity within certain regions of the DMN such as the posterior cingulate and precuneus.

A slightly unexpected finding was that BMI and BFP were positively correlated with global gray matter volume (*p* = 0.07 and *p* = 0.04, respectively) and negatively correlated with global CSF volume (*p* = 0.08 and *p* = 0.07, respectively), corrected for age, gender, and TICV (Figure S2). These findings are not consistent with previous reports, which have traditionally suggested that BMI is negatively correlated with global brain and parenchymal volumes (Ward et al., 2005; Gunstad et al., 2008; Debette et al., 2010; Cazettes et al., 2011; Bobb et al., 2014). However, since some of these studies corrected for TICV and some did not, we also performed global tissue volume analyses on our data without correcting for TICV.

After correcting for age and gender only, global gray matter volume was even more positively correlated with both BMI and BFP (*p* = 0.001 and *p* = 0.007, respectively), global white matter volume was positively correlated with BMI but not BFP (*p* = 0.04 and *p* = 0.23, respectively), global CSF volume was not significantly correlated with either BMI or BFP (*p* = 0.48 and *p* = 0.31, respectively), and TICV itself was positively correlated with BMI and BFP (*p* = 0.01 and *p* = 0.07, respectively). Therefore, in contrast to our regional gray and white matter volume results (Figure 3 and Table 1)—which support and extend previous findings linking elevated BMI to decreased regional gray and white matter volumes—our global tissue volume analyses indicate that overall brain and parenchymal volumes are not necessarily decreased, and may in fact be increased, as a function of BMI and BFP (regardless of whether or not individual differences in TICV were corrected for).

Although network-based white matter analyses—i.e., from either anatomically-guided or fMRI-guided “connectome reconstructions” of white matter regions belonging to each network (as reported in the current study)—have enormous potential for investigating quantitative white matter differences, only one other study has very recently been published using similar methods to investigate white matter correlates of body composition (Marqués-Iturria et al., 2015). In their study, the authors investigated an ostensive white matter reward system (comprised of the anatomical connections between bilateral lateral orbitofrontal, medial orbitofrontal, caudate, putamen and nucleus accumbens regions), finding both reduced numbers of DTI tractography streamlines and lower FA values in obese subjects compared to healthy-weight controls. These findings are consistent with many of the negative associations between body composition and both white matter volume (Figure 3) and microstructure (Figure 4) observed in the current study, and our work further suggests that there are volumetric differences throughout the DMN, ECN, and SN (Figure 5). Although it has been suggested that high-fat diets could increase apoptosis (Watson et al., 2000; Moraes et al., 2009), and this may partially explain the volumetric differences observed in our study, it is more likely that lower brain volumes in subjects with higher BMI and BFP can be explained by decreased neurogenesis (Lindqvist et al., 2006), lower dendritic spine density (Baran et al., 2005), or other cellular or molecular factors influencing brain volume.

### Increased functional connectivity in the face of decreased structural connectivity

Our structural imaging data indicate that individuals with higher BMI and/or BFP exhibit significant gray and white matter volume reductions (Figure 3 and Table 1), as well as widespread changes in white matter microstructure (Figure 4 and Table 2) surrounding the dorsal striatum and regions of the DMN, ECN, and SN. In light of previous studies reporting positive structure-function relationships (see Honey et al., 2010 for review), identifying such widespread decreases in structural connectivity (i.e., white matter volume and microstructure) seems to conflict with the observation of increased functional connectivity. However, findings similar to ours have been reported in healthy aging, where elderly subjects were found to compensate for lower white matter integrity with increased neural activity (Daselaar et al., 2015), and among Multiple Sclerosis (MS) patients, where significant decreases in white matter integrity have been associated with reduced cognitive performance (as expected), but also with increased resting state functional connectivity in the DMN and ECN (Hawellek et al., 2011). Although the basis of these anti-correlations (i.e., between structural and functional connectivity) has not been confirmed, one plausible explanation is that communication between regions is occurring via alternate pathways. The functionally-defined white matter atlases from which quantitative white matter measures were extracted (Figley et al., 2015) were generated separately for dorsal and ventral portions of the DMN, for left and right components of the ECN, and for anterior and posterior components of the SN. Therefore, the inter-network connections (e.g., between dorsal and ventral DMN) are not accounted for in our ROI-based white matter analyses. However, another plausible explanation is that increased functional connectivity within a network may be somewhat of a compensatory mechanism for reduced structural connectivity. For example, if the quality of information transmission is poor between brain regions (e.g., due to reduced anatomical connectivity), the functional communication may be enhanced (e.g., by increased neuronal firing rates and/or temporal synchrony) to increase the efficiency of communication. Indeed, electrophysiological studies in rodents show that the reduction of afferent fibers after normal aging is met with an increase in synaptic field potentials in the remaining fibers (Barnes and McNaughton, 1980; Burke and Barnes, 2010).

### Implications for cognition, impulsivity, and salience/reward processing

Having established that individual differences in body composition are *associated* with structural and functional brain changes does not address questions regarding the *causes* and *effects* of these changes and their relation to cognition, reward processing, or impulsivity. As previously mentioned, several lines of research have shown that obesity is associated with lower scores on decision-making and executive function tasks (Gunstad et al., 2007, 2010; Verdejo-García et al., 2010; Maayan et al., 2011; Mobbs et al., 2011), compromised inhibitory control (Waldstein and Katzel, 2006; Verdejo-García et al., 2010; Maayan et al., 2011; Mobbs et al., 2011), slower mental processing (Cournot et al., 2006; Waldstein and Katzel, 2006; Gunstad et al., 2007; van den Berg et al., 2009), and decreased learning and semantic memory performance (Elias et al., 2003; Cournot et al., 2006; Nilsson and Nilsson, 2009; Gunstad et al., 2010). However, are the types of structural and functional brain differences observed in our study likely to *cause* such decreases in cognitive performance and self-control? And if so, might these neural differences further predispose certain individuals to poorer body composition? Or are these differences more likely to be an *effect* of poor diet, lifestyle, and/or body composition?

Although our study does not directly answer these questions, interpreting the present findings in the context of previous literature may provide some insight. For example, previous studies suggest that the kinds of widespread functional and structural brain differences observed in our study might: (1) *cause* or exacerbate previously observed cognitive and behavioral deficits among overweight individuals, and (2) promote obesity by diminishing the capacity of high BMI and BFP participants to establish and maintain healthy lifestyle choices. In particular, the SN is known to play a significant role in emotional control (Seeley et al., 2007), cognitive control (Menon and Uddin, 2010), error processing (Ham et al., 2013), regulating the balance between DMN and ECN activity (Bressler and Menon, 2010), and switching between exogenous and endogenous attentional states (Bressler and Menon, 2010).

Prior work with patient populations has shown that decreased cognitive processing and inhibitory control following traumatic brain injury (TBI) are associated with impaired white matter integrity in the SN (Bonnelle et al., 2012), and that early frontotemporal dementia specifically affects regions of the SN (Seeley et al., 2006). Gray matter volumes in the SN are also significantly reduced in both Alzheimer's disease (AD) and mild cognitive impairment (MCI) patients compared to healthy controls, and these volume reductions correspond with decreased cognitive performance (He et al., 2014). In this regard, our data showing that body composition has widespread correlations with gray and white matter volume reductions (Figure 3)—with significant negative associates with white matter volume throughout the DMN, ECN, and SN (Figure 5)—appears to be in line with previous reports linking obesity to lower cognitive performance, and possibly even dementia (Whitmer et al., 2008; Naderali et al., 2009).

Furthermore, high-fat diets and obesity are linked to impulsivity and self-control deficits (Gunstad et al., 2007; Maayan et al., 2011; Sutin et al., 2011; Jasinska et al., 2012), and one compelling study has recently implicated the SN, in particular, as the hub for controlling motivation, willpower, and the ability to persevere through physical and/or emotional challenges (Parvizi et al., 2013). Taken in this context, the fact that elevated BMI and BFP in our study were related to aberrant SN function (Figure 2) and structure (Figure 5) is consistent with the observation that many overweight individuals appear to have diminished resolve in terms of altering their lifestyle habits (e.g., diet and exercise), and with previous findings that obese participants show greater hedonic responses to food in gustatory and somatosensory cortices, along with reduced caudate activation, compared to their leaner counterparts (Stice et al., 2008).

It has also been reported that striatal dopamine D_2_ receptor densities are decreased among obese subjects (Wang et al., 2001; Volkow et al., 2008); and, since these dopamine circuits modulate pleasure and reward processes, lower dopaminergic signaling is thought to perpetuate addictive behaviors (including poor diet and overeating) in order to compensate for lower than normal pleasure responses—a phenomenon known as *Reward Deficiency Syndrome* (c.f. Blum et al., 2006, 2014). Our findings of reduced gray and white matter volumes (Figure 3 and Table 1) and reduced white matter integrity (Figure 4 and Table 2) throughout striatal (dopeminergic) regions, appear to be consistent with this notion; and it is important to distinguish that higher functional connectivity within the SN (Figure 2) among higher BMI and BFP individuals does not imply more dopeminergic activity or salience processing in response to food or other stimuli. It simply means that the regions comprising this network have a higher degree of covariance at rest, which (as described above in the Section on “Increased Functional Connectivity in the Face of Decreased Structural Connectivity”) may even be indicative of network impairment.

However, although differences in brain structure and function (as reported in the current study) might reduce self-control and alter reward processing in ways that lead to poor diet, etc., it has been suggested that the reverse may also hold true—i.e., where unhealthy lifestyle choices and body composition might also affect neurobiology and behavior. For example, Western diets that are high in saturated fat and processed sugar appear to cause deficits related to energy regulation and promote continued food consumption despite satiety signals (Davidson et al., 2007; Kanoski and Davidson, 2011). Previous work with animal models has also shown that high-fat diets and obesity cause increased oxidative stress and neuroinflammation (White et al., 2009; Pistell et al., 2010), increased blood-brain barrier permeability (Kanoski et al., 2010), reduced neurogenesis (Lindqvist et al., 2006), lower levels of brain-derived neurotrophic factor (BDNF) (Molteni et al., 2002; Kanoski et al., 2007), and both decreased dendritic integrity and synaptic plasticity (Granholm et al., 2008; Stranahan et al., 2008). Interestingly, obese rodents receiving a high-fat diet have significantly increased dopamine receptor densities throughout the nucleus accumbens, caudate, and putamen (South and Huang, 2008)—i.e., areas in which we observed significant volumetric and microstructural changes, and which are known to be critically involved in reward processing, decision-making, and habit learning (Nicola et al., 2000; Balleine et al., 2007). These and other data clearly indicate that diet and body composition have measurable effects on the brain, down to the cellular and molecular level.

Taken together, previous human and animal work suggest that the complex relationships between obesity, brain structure and function (such as the results reported in the current study), and cognitive performance may be cyclical—such that poor diet and high amounts of body fat could *cause* structural and functional brain changes that suppress self-control and increase food consumption, which (in turn) promotes further increases in body mass and body fat. However, given the cross-sectional nature of the current study, further research involving longitudinal and/or animal experiments will be required to confirm and further explain the complex cause and effect relationships between obesity, altered brain structure and function (particularly throughout the DMN, ECN, and SN), cognitive deficits, and the ability to make lasting lifestyle changes.

### Limitations of the current study

Although not “small” compared to most fMRI, VBM, and/or DTI studies, one of the primary limitations of this study is the relatively low sample size compared to many behavioral and epidemiological data sets. After correcting for age and gender effects (thereby reducing the degrees of freedom) and correcting for multiple comparisons, many of the partial correlations shown in Figures 2,5–7 that were marginally significant (e.g., *p*_adj._ ≈ 0.10) may have become more significant if the sample size was slightly larger. However, due to practical considerations (including the high cost of MRI scanning relative to the overall budget for this project) that precluded recruiting more subjects, several measures were taken in an attempt to optimize our study design and data analyses. Perhaps the most significant of these was the implementation of a regression-based analysis approach in order to capitalize on individual differences (as opposed to binning subjects into categories and performing a more conventional group-wise analysis). Capturing variance in this way was highly effective, and should be considered in future analyses of similar data sets, regardless of sample size.

Another possible limitation of the current study was that we did not acquire detailed neuropsychological measures to assess subject-specific measures of executive function, impulsivity, etc. within our sample. However, although it might have been ideal to correlate BMI and BFP with individual differences in cognitive performance among our study-specific population (and possibly correlate these with each of the neuroimaging measures), we felt that there was already overwhelming evidence—based on several of the large-scale and/or longitudinal studies referenced above—for cognitive changes associated with obesity, and therefore focused our efforts on neuroimaging data acquisition and analysis.

## Conclusions

Negative associations between obesity, physical health, and life expectancy are common knowledge, but more recent studies have also shown that, on average, being overweight: (1) reduces attention, working memory, executive function, and inhibitory control among otherwise healthy participants, and (2) increases the risk of developing early onset dementia. The current experiment employed advanced neuroimaging techniques and two different body composition measures to establish possible mechanisms for obesity-related cognitive, behavioral, and neural differences among healthy adults. The neural systems that showed consistent associations with body composition (i.e., across multiple functional and structural neuroimaging modalities) offer a biologically plausible mechanism for reduced cognitive performance and self-control among overweight individuals. In particular, our results indicate that higher BMI and BFP are associated with increased functional connectivity, decreased regional gray and white matter volumes, and decreased white matter microstructure throughout regions and networks known to subserve cognitive, salience, and reward processing functions. Therefore, interpreted in the context of previous research, these findings suggest that changes in body composition may be cyclically (causally) related to alterations in brain structure and function that could decrease self-control and promote further unhealthy behavior.

## Author contributions

CF was involved in all aspects of the project, including its conception, study design, data acquisition, data analysis, data visualization, data interpretation, and manuscript preparation. JA was involved in project conception, study design, data acquisition, data interpretation, and manuscript preparation. EL was involved in project conception, study design, data acquisition, data interpretation, and manuscript preparation. SC was involved in project conception, study design, data acquisition, data interpretation, and manuscript preparation.

### Conflict of interest statement

The authors declare that the research was conducted in the absence of any commercial or financial relationships that could be construed as a potential conflict of interest.

## Footnotes

^1^For your convenience, please refer to the List of Abbreviations in the Supplementarily Material.

^2^Based on previous literature (Kullmann et al., 2012), our one-tailed *t*-tests assumed that positive correlations would be found between body composition and DMN connectivity. Therefore, because the correlations were actually slightly negative in our sample, the significance levels were extremely low for both BMI and BFP (Figure 2B). Nonetheless, even if we had assumed negative correlations, the resulting one-tailed *t*-tests would not have yielded significant correlations after comparing for multiple comparisons (*p*_adj._ = 0.43 and *p*_adj._ = 0.39, respectively), so this finding does not strongly affect the overall conclusions.
