# Supplementary figures and images for: Effects of Body Mass Index and Body Fat Percent on Default Mode, Executive Control, and Salience Network Structure and Function
Source: Front Neurosci. 2016 Jun 14;10:234. doi: 10.3389/fnins.2016.00234 (PMC4906227; doi:10.3389/fnins.2016.00234)

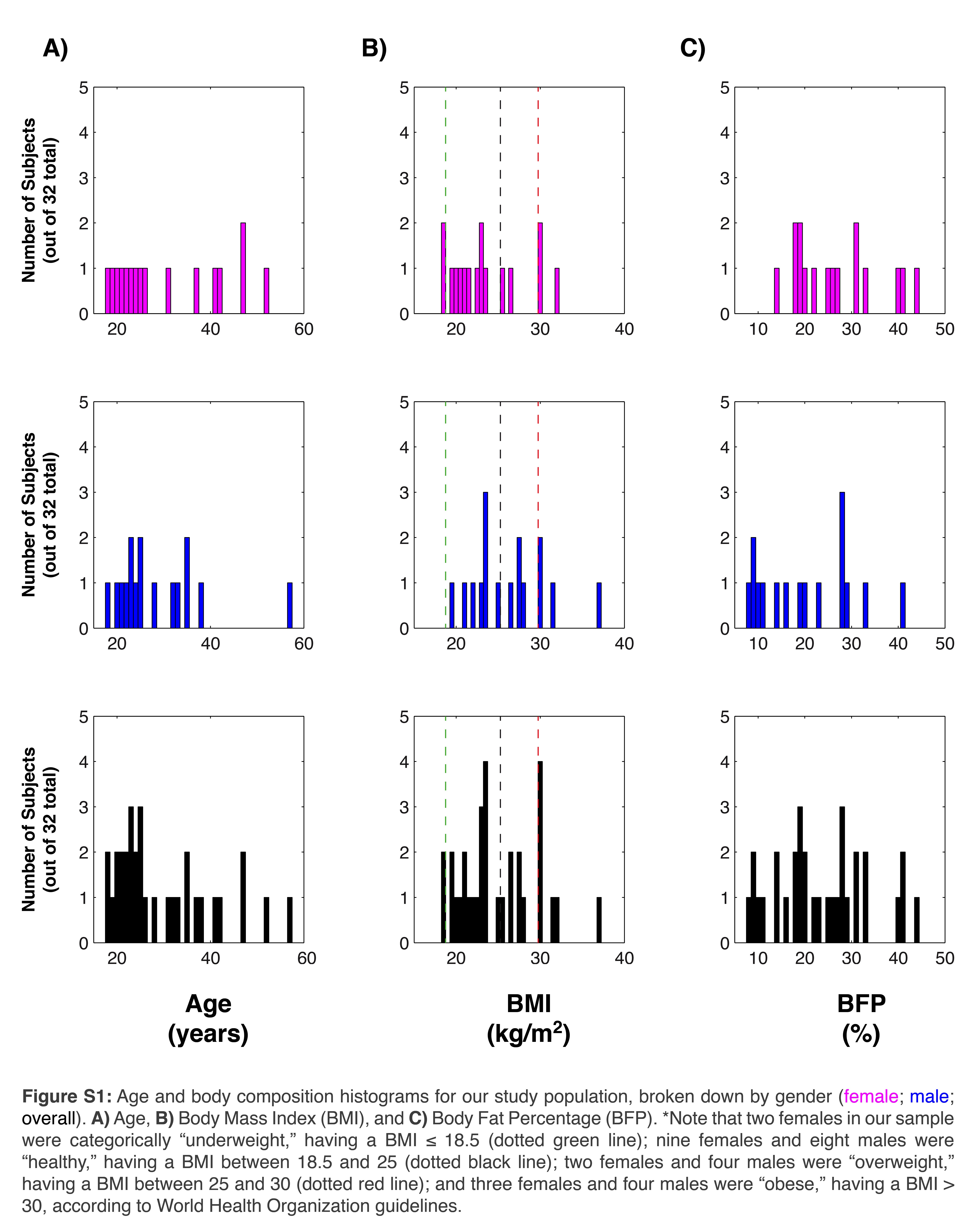

Supplement: Supplementary file 1 [file FigureS1.JPEG]

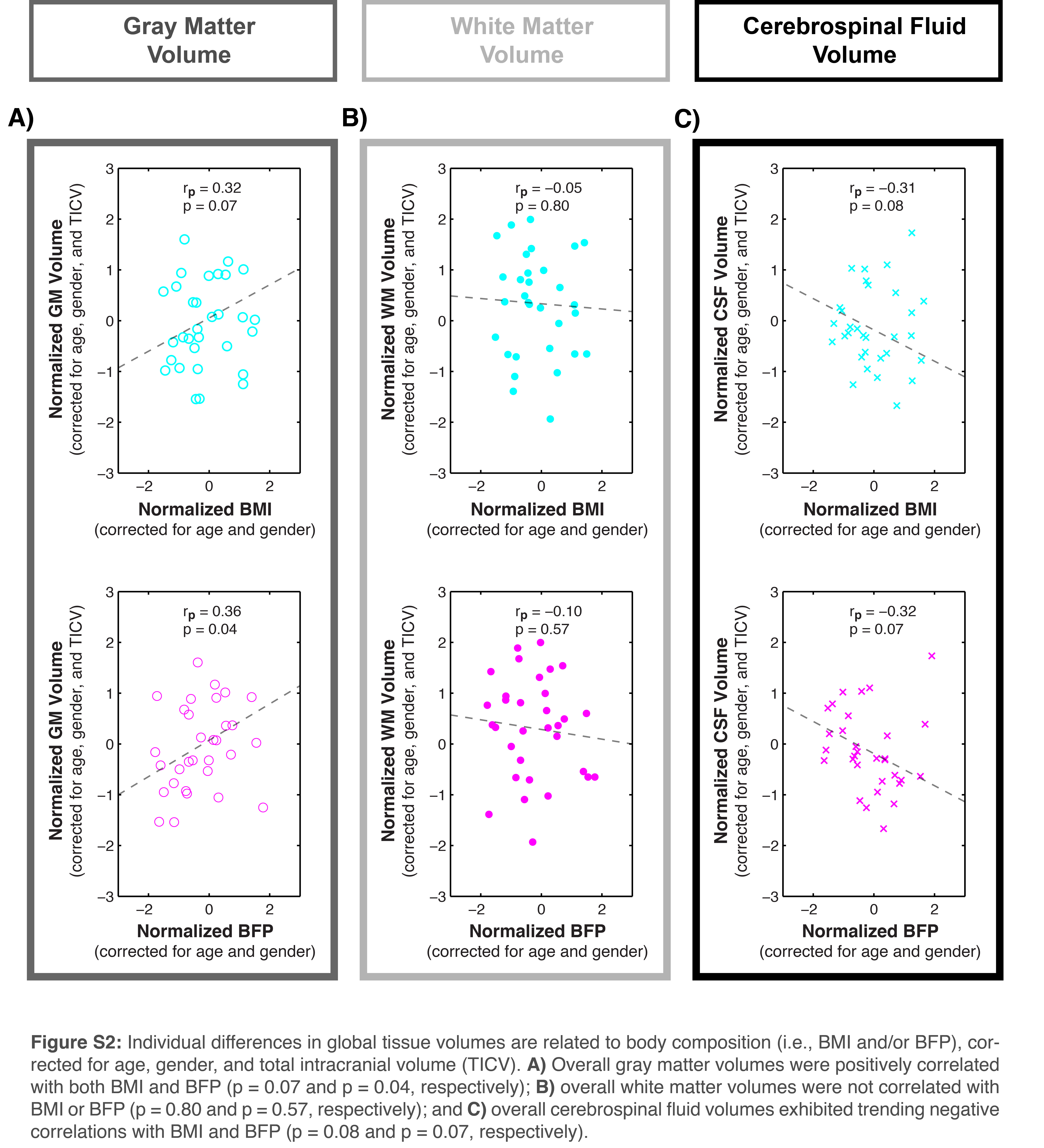

Supplement: Supplementary file 2 [file FigureS2.JPEG]

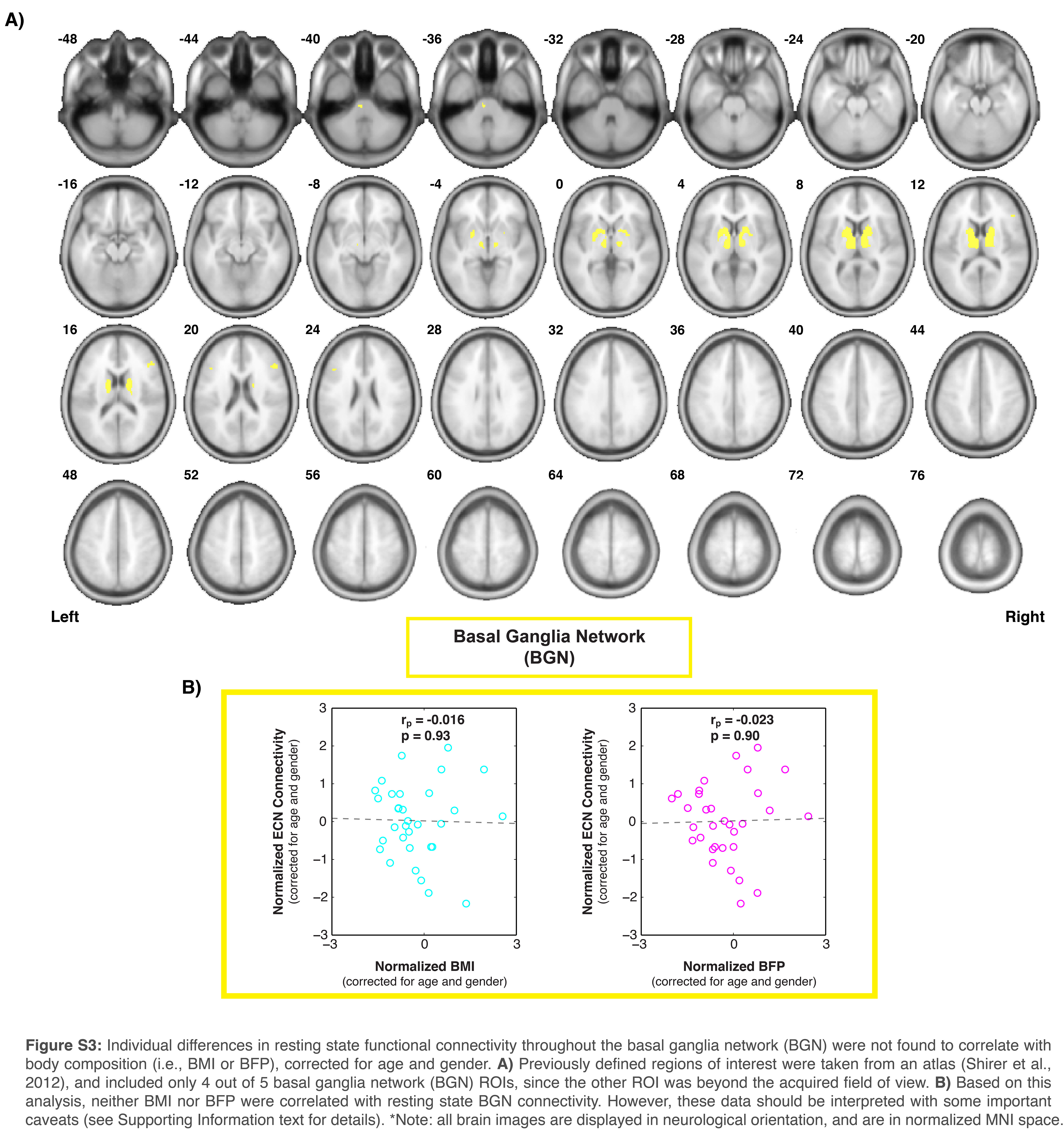

Supplement: Supplementary file 3 [file FigureS3.JPEG]
