# Supplementary material for: Effects of Body Mass Index and Body Fat Percent on Default Mode, Executive Control, and Salience Network Structure and Function
Source: Front Neurosci. 2016 Jun 14;10:234. doi: 10.3389/fnins.2016.00234 (PMC4906227; doi:10.3389/fnins.2016.00234)
Supplement: Supplementary file 4 [file ListofAbbreviations.PDF]

List of Abbreviations

AP = Anterior to Posterior (Direction)

BDNF = Brain-Derived Neurotrophic Factor

BFP = Body Fat Percent

BGN = Basal Ganglia Network

BIA = Bioelectric Impedance Analysis

BMI = Body Mass Index

CSF = Cerebrospinal Fluid

DARTEL = Diffeomorphic Anatomical Registration Through Exponentiated Lie Algebra

DEXA = Dual-Energy X-Ray Absorptiometry

DMN = Default Mode Network

DTI = Diffusion Tensor Imaging

ECN = Executive Control Network

FA = Fractional Anisotropy

FDR = False Discovery Rate

FH = Feet to Head (Direction)

fMRI = Functional Magnetic Resonance Imaging

FWE = Family-Wise Error

FWHM = Full-Width at Half-Maximum

GE-EPI = Gradient Echo, Echo Planar Imaging

GM = Gray Matter

ICBM = International Consortium for Brain Mapping

IRB = Institutional Review Board

JHU = Johns Hopkins University

LDDMM = Large Deformation Diffeomorphic Metric Mapping

MD = Mean Diffusivity

MNI = Montreal Neurological Institute

MRI = Magnetic Resonance Imaging

RL = Right to Left (Direction)

ROI = Region of Interest

rs-fMRI = Resting State Functional Magnetic Resonance Imaging

SE-EPI = Spin Echo, Echo Planar Imaging

SN = Salience Network

TE = Echo Time

TICV = Total Intracranial Volume

TR = Repetition Time

VBM = Voxel Based Morphometry

WM = White Matter
