# Supplementary material for: Effects of Body Mass Index and Body Fat Percent on Default Mode, Executive Control, and Salience Network Structure and Function
Source: Front Neurosci. 2016 Jun 14;10:234. doi: 10.3389/fnins.2016.00234 (PMC4906227; doi:10.3389/fnins.2016.00234)
Supplement: Supplementary file 5 [file SupportingInformation.PDF]

SUPPORTING INFORMATION

Assessing Functional Connectivity within the Basal Ganglia Network

Given that we observed such widespread volumetric and microstructural changes throughout the dorsal striatum (Figures 3-5), we decided to investigate whether or not any obvious correlations between body composition (BMI and BFP) and intrinsic basal ganglia network (BGN) connectivity could be found. Similar to our previous rs-fMRI analyses, ROIs for the BGN were taken from an *a priori* atlas of functionally connected brain regions (Shirer *et al.*, 2012). Based on this atlas, the BGN is crudely segmented compared to other networks and only consists of 5 ROIs (Figure S3A); because one of these ROIs (in the inferior brainstem; shown in slices z = -36 and z = -40) was below the acquired field-of-view of the rs-fMRI for most of our participants, this analysis was further limited to include only the 4 remaining regions.

Compared to the 19 DMN regions, 12 ECN regions, and 19 SN regions, including so few regions is expected to affect the average network connectivity scores by increasing the sensitivity to noise or outliers in only a small number of regions. Moreover, this problem is likely to be exacerbated because the image processing pipeline for the rs-fMRI data included the standard, linear SPM8 normalization (as described in the main text Methods) – as opposed to the non-linear DARTEL or LDDMM normalization approaches used in our VBM or DTI analyses, which perform much better for aligning sub-cortical structures. Using the linear registration approach, it is likely that two (half) of the remaining ROIs located in the left and right sub-cortical regions are not optimally co-registered across participants. Consequently, re-normalizing all of the rs-fMRI data (222 volumes for all 32 participants) goes beyond the scope of this paper. Nonetheless, with these caveats in mind, we did not observe any correlations between BGN connectivity and either BMI or BFP ($p = 0.93$ and $p = 0.90$, respectively) among our sample population, corrected for individual differences in age and gender (Figure S3B).
